# Difference between Selenite and Selenate in the Regulation of Growth and Physiological Parameters of Nickel-Exposed Lettuce

**DOI:** 10.3390/biology9120465

**Published:** 2020-12-12

**Authors:** Barbara Hawrylak-Nowak, Renata Matraszek-Gawron

**Affiliations:** Department of Botany and Plant Physiology, Faculty of Environmental Biology, University of Life Sciences in Lublin, Akademicka 15, 20-950 Lublin, Poland; renata.matraszek@up.lublin.pl

**Keywords:** metal toxicity, selenium, nickel, abiotic stress, lipid peroxidation, root viability

## Abstract

**Simple Summary:**

Nickel is a trace metal that can cause toxicity in plants. In contrast, selenium, as a beneficial element, can have a favorable effect on plants grown under metal stress. However, the biological activity of selenium largely depends on its chemical form. Therefore, we assessed the effect of two chemical forms of Se (selenite and selenate) on the toxicity and accumulation of Ni in lettuce. We found that Ni phytotoxicity severely increased in the presence of higher doses of selenite. On the other hand, a low dose of selenate stimulated the growth of roots of Ni-exposed plants. We found that selenium can modify Ni activity in lettuce, but this depends not only on the concentration but also on the form of selenium. The strong intensification of Ni toxicity in the presence of selenite was most likely related to the very high selenium accumulation in root tissues. The obtained results indicate that depending on the conditions in the root environment, Se in a given concentration and chemical form can be either beneficial or toxic for plants. Therefore, the use of selenium in metal-stressed plants requires consideration of not only the concentration but also the chemical form of this element.

**Abstract:**

Nickel is an essential plant micronutrient; however, even at low concentrations, it may be phytotoxic. Selenium is a beneficial element with an alleviating effect that has been confirmed in the case of many abiotic stresses, including metal toxicity. The aim of this study is to assess the effect of two forms of Se (Se(IV) or Se(VI)) on the phytotoxicity, accumulation, and translocation of Ni in lettuce. Nickel causes a reduction in lettuce growth and vitality of roots, probably through increased lipid peroxidation. The application of Se(IV) to a Ni-contaminated medium resulted in a further reduction of growth, especially in the presence of 6 µM Se(IV). The growth-promoting effect of Se was found only in the 2 µM Se(VI)/10 µM Ni treatment. The application of 6 µM Se, regardless of the Se form, to the Ni-containing substrate caused an increase in shoot Ni concentration. In turn, a decrease in root Ni content was found for all Se treatments. The strong aggravation of Ni phytotoxicity in the presence of 6 µM Se(IV) was most likely related to the accumulation of high Se concentration in the roots, and the combination of high root Ni accumulation caused irreversible dysregulation of cell metabolism.

## 1. Introduction

Nickel is classified as a plant micronutrient since it is a component of the active site of urease, i.e., the only known Ni-containing plant enzyme [1]. However, besides their ureolytic activity, ureases may also be involved in defense functions in plants [2]. Recently, it has been shown that Ni ions may activate glyoxalase I isoenzyme, which participates in the degradation of cytotoxic methylglyoxal (MG) [3]. Methylglyoxal is a highly reactive compound produced naturally in cells through both enzymatic and nonenzymatic reactions, especially under stress. However, excess Ni produces phytotoxic effects [4,5]. The physiological background of the phytotoxicity of Ni ions is not entirely clear. The two indirect mechanisms of Ni phytotoxicity are (i) induction of oxidative stress and (ii) interaction with other essential nutrients [1,5]. 

Although Ni concentrations in the environment rarely exceed legislative limits, this metal, due to its high mobility and bioaccumulation ability, may represent a serious hazard to humans, animals, and plants [6]. The toxicity of Ni-containing materials is considered to be related to the bioavailability of the ionic form of the metal (Ni^2+^). Ni sources from both natural (volcanic eruptions, soil dust, forest fires, vegetation exudates) and human activities (emissions from smelting and refining processes, fossil fuel combustion, disposal of sewage sludge, or application of sludge as a fertilizer) may contribute to Ni loading in terrestrial and aquatic environments [7]. Since the level of Ni pollution has substantially increased in recent decades, mainly due to anthropogenic activity [8,9], it is important to find effective methods to alleviate its toxic effects on plants. 

Many studies on the effect of Se as a beneficial element for plants have shown that it can effectively mitigate the negative effects of environmental stresses, especially the consequences of oxidative stress [10,11]. The latest results on the effects of Se supplementation on metal-exposed plants indicate that coprecipitation of Se and metals in soil plays a key role in inhibiting the uptake of metals by plants. Selenium may also affect metal uptake through the modification of root morphology by the action of phytohormones [12], an increase in the proportion of less mobile metal complexes, and better metal binding to the root cell wall as a result of elevated amounts of pectin and hemicellulose, all of which lead to a limitation of metal transport across the plasma membrane [13]. Therefore, in the presence of Se, restricted Cd accumulation was observed in fruits [14] and seeds [15,16]. Application of Se under Cd stress caused an increase in catalase (CAT), superoxide dismutase (SOD), ascorbate peroxidase (APX), and glutathione reductase (GR) activities, inducing more efficient scavenging of reactive oxygen species (ROS). Similarly, accumulation of osmolytes, i.e., proline and glycine betaine, was enhanced by Se supplementation [17]. The roots of Cd-exposed plants treated with Se were characterized by a reduced level of lipid peroxidation as well as decreased Cd and phytochelatin (PC) concentrations in comparison to Cd treatment alone, while these parameters were not affected in shoots [18]. In our previous study, we investigated the effects of Se(IV) on lettuce exposed to 50 or 100 µM Ni [19,20]. We obtained positive results of Se supplementation on plant growth and accumulation of macronutrients at a concentration of 5 µM, with an ambiguous effect on the content of Ni in leaves, which was Ni and Se dose-dependent. A concentration of 20 µM Se(IV) was already toxic to plants. In all the studies cited above, Se was most often used in its two inorganic soluble forms: Se(IV) or Se(VI). Although Se has a narrow physiological window and its beneficial effects or toxicity largely depends on Se species [21], the comparative impact of these two Se forms on plants subjected to metal stress has so far been studied only in Cd-exposed *Brassica chinensis* [22,23]. The effect of Se application at various concentrations and chemical forms has not been studied in Ni-stressed plants to date. For this reason, we decided to conduct such research by additionally analyzing root and shoot Ni and Se content and their translocation, root viability, and root lipid peroxidation in young lettuce plants (2 weeks old). Moreover, the Ni concentrations used here (5 or 10 µM Ni) were in the range of concentrations noted in the water of contaminated industrial regions [24,25] and were much lower than those used in our previous experiments [19,20].

The hypothesis that Se(IV) and Se(VI) differently modulate the physiological response of Ni-stressed lettuce and affect the accumulation and translocation of Ni was tested. The objectives of this study are to (i) compare the effects of two concentrations and two forms of Se on the growth and selected physiological parameters of lettuce, (ii) compare the effect of Se application on plants grown at two Ni concentrations, and (iii) investigate the influence of Se treatments on the bioconcentration and translocation of Ni and Se. 

## 2. Materials and Methods

### 2.1. Plant Growth Conditions and Experimental Treatments

Lettuce (*Lactuca sativa* L.) was selected as a potential bioindicator species that is recommended for the assessment of phytotoxicity of industrial contaminants containing trace metals [26]. Seeds of butter-head lettuce (*Lactuca sativa* var. *capitata* L.) cv. Justyna were sown onto moist quartz sand and allowed to germinate. Germination took place in a laboratory at 20–23 °C for about 14 days. Three best-developed seedlings of uniform size were placed in glass jars (1 L) filled with a 1.5-fold strength Hoagland II nutrient solution supplemented with micronutrients (pH = 6.0) [27]. The medium was then differentiated in terms of the concentration of Ni (0, 5, or 10 µM as NiCl_2_) and Se (2 or 6 µM applied as Na_2_SeO_3_ or Na_2_SeO_4_). The concentrations of Ni were chosen on the basis of preliminary experiments. The Se(IV) and Se(VI) concentrations were selected as those with a proven growth-stimulating effect on lettuce cv. Justyna [28]. The experiment consisted of 11 treatments with 4 replications per each treatment and was independently repeated twice in the same conditions to ensure the authenticity of the results. The following Ni and Se treatments were used: the control (0 Ni/0 Se); 5 μM Ni; 5 μM Ni + 2 µM Se(IV); 5 μM Ni + 6 µM Se(IV); 5 μM Ni + 2 µM Se(VI); 5 μM Ni + 6 µM Se(VI); 10 μM Ni; 10 μM Ni + 2 µM Se(IV); 10 μM Ni + 6 µM Se(IV); 10 μM Ni + 2 µM Se(VI); 10 μM Ni + 6 µM Se(VI). The plants were grown in a controlled climate growth chamber (Sanyo, MRL 350 HT) with a photosynthetic photon flux density (PPFD) of 250–270 µmol m^−2^ s^−1^, 14 h photoperiod, the temperature of 22/18 °C (day/night), and 60–65% relative humidity.

After 14 days of growth in the differentiated experimental conditions, the plants were harvested and analyzed for physiological and biochemical parameters. The remaining plant material (roots and shoots separately) was dried at 80 °C to a constant weight, ground, and analyzed for Ni and Se concentrations.

### 2.2. Determination of Growth Parameters and Concentration of Photosynthetic Pigments 

The aboveground organs were cut from the roots for the determination of their fresh weight (FW). The second pair of true leaves were excised from each plant and scanned for leaf area (LA) using a CI-202 laser area meter (CID Bio-Science, Camas, WA, USA). 

The concentrations of chlorophyll a, b, and carotenoids were measured in the second pair of true leaves by homogenization of the leaf samples with 80% (*v*/*v*) aqueous acetone. The extracts were filtered using a vacuum pump. The absorbance of the samples was measured spectrophotometrically (Cecil CE 9500, Cecil Instruments, Cambridge, UK) at 663, 646, and 470 nm. The concentrations of photosynthetic pigments were calculated from equations proposed by Lichtenthaler and Wellburn [29].

### 2.3. Measurement of Lipid Peroxidation 

The intensity of lipid peroxidation in leaf (the second pair of true leaves) and root tissues was quantified by measuring the concentration of thiobarbituric acid reactive substances (TBARS) using the methods described in detail previously [28,30]. An extinction coefficient of 155 mM^−1^ cm^−1^ was used for the calculation of TBARS concentrations.

### 2.4. Visualization of Root Viability with the TTC Method

A 2,3,5-triphenyltetrazolium chloride (TTC) dye is commonly used as an indicator of cellular respiration and cell viability. Its action is based on the principle that all living cells contain active dehydrogenases that catalyze chemical reductions. This method is based on visual detection of dehydrogenase activity in root tips using 2,3,5-triphenyltetrazolium chloride (TTC) [31]. Lettuce root tips (about 10 mm long) were incubated with a 0.6% (*w*/*v*) TTC in 0.06 M Na_2_HPO_4_/KH_2_PO_4_ buffer (pH = 7.4) containing 0.05% (*v*/*v*) Tween 20 for 30 min. Under the influence of dehydrogenases, the colorless TTC is transformed into red–pink triphenylformazan (TPF). The microscopic preparations were observed in visible light under a microscope (Carl Zeiss Axioskop 20, Jena, Germany), and images were captured using a digital camera (Nikon DS-Fi1, Nikon Inc., Tokyo, Japan). 

### 2.5. Determination of Ni and Se Concentrations

To remove cell-wall–bound Ni^2+^, the lettuce roots were desorbed in a 50 mM CaCl_2_ solution (*w*/*v*) for 5 min, rinsed in distilled water, and blotted on filter paper. Then, the roots and aboveground parts were dried. The root and shoot dry materials were mineralized in a mixture of HNO_3_–HClO_4_ acids (4:1; *v*/*v*) at 210 °C. The Ni concentrations were determined with classic atomic absorption spectroscopy (AAS; GBC Avanta, Sigma, St. Louis, MO, USA). Hydride generation atomic absorption spectroscopy (HG-AAS) was used to determine total Se concentrations, as described previously [28]. 

### 2.6. Statistical Analyses

The data collected were subjected to one–way analysis of variance (ANOVA) with Tukey’s posthoc test at the 0.05 probability level. To conduct statistical analysis, Statistica ver. 13.3 software (TIBCO Software Inc., Palo Alto, CA, USA) was used. The images of root viability were collected from at least ten root tips per treatment. 

## 3. Results

### 3.1. Effect of Se(IV) and Se(VI) on the Growth and Content of Photosynthetic Pigments in Ni-Exposed Lettuce

**Figure 1:**
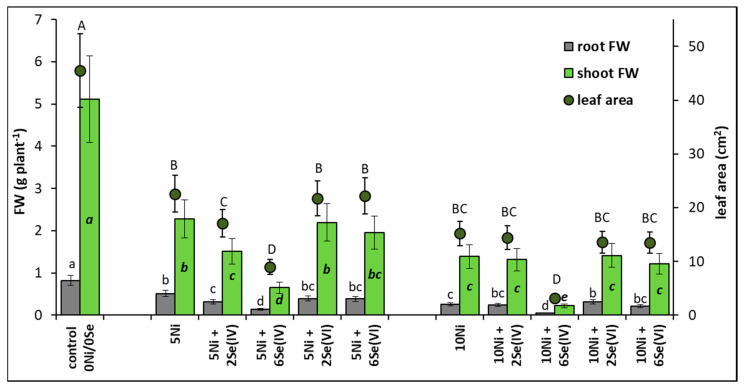
Effect of Se (selenite and selenate) supplementation on the growth parameters of Ni-exposed lettuce after 14 days of exposure. Data are means ± SD (*n* = 24). Means for individual organs followed by the same letter are not significantly different (*p* < 0.05, Tukey’s test).

The plant growth parameters analyzed are presented in Figure 1. In the presence of 5 or 10 µM Ni, shoot FW decreased by 54% or 73%, and root FW decreased by 35% or 69%, respectively, in comparison to the control plants. Ni-induced reduction of leaf area was also found. The addition of 2 or 6 µM Se(IV) to the medium containing 5 µM Ni contributed to a further decrease in shoot FW by 34% and 72% and root FW by 39% and 73%, respectively, in comparison to plants grown at 5 µM Ni alone. It was accompanied by a significant reduction of leaf area. A severe reduction in shoot and root FW (by 82–84%) was also found in the presence of 6 µM Se(IV) in the medium containing 10 µM Ni. In contrast, the application of Se(VI) did not generally cause significant changes in plant FW and leaf area, in comparison to plants grown in the Ni-alone treatments. An exception was the significant increase in root FW (by 23%) after supplementing the nutrient solution containing 10 µM Ni with 2 µM Se(VI). 

**Figure 2:**
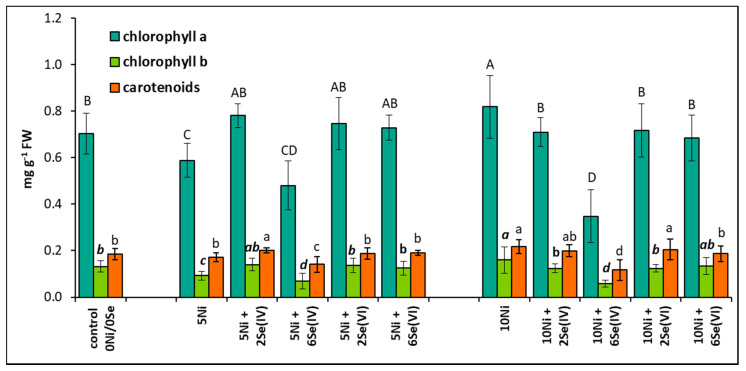
Effect of Se supplementation on the concentration of photosynthetic pigments in Ni-exposed lettuce after 14 days of exposure. Data are means ± SD (*n* = 6). Means for individual pigment classes followed by the same letter are not significantly different (*p* < 0.05, Tukey’s test).

The concentration of chlorophyll a and b decreased by 16% and 30%, respectively, in plants treated with 5 µM Ni but increased by 17% and 21%, respectively, in those exposed to 10 µM Ni, compared to the control (Figure 2). The application of 6 µM Se(IV) to the Ni-containing medium in general significantly decreased the accumulation of photosynthetic pigments, especially at the higher Ni dose. In these conditions, the content of chlorophyll a, b, and carotenoids decreased by 57%, 63%, and 46%, respectively, compared with the Ni-alone treatments. Conversely, the application of 2 µM Se(IV) and Se(VI) at both concentrations positively influenced the accumulation of photosynthetic pigments in lettuce exposed to 5 µM Ni. The opposite effect of Se on the bioaccumulation of pigments was found at the higher concentration of Ni. However, under Se supplementation (except for 6 µM Se(IV)), the level of chlorophylls was similar to their concentrations in the leaves of the control plants (Figure 2).

### 3.2. Effect of Se(IV) and Se(VI) on the Level of Lipid Peroxidation in Ni-Exposed Lettuce

**Figure 3:**
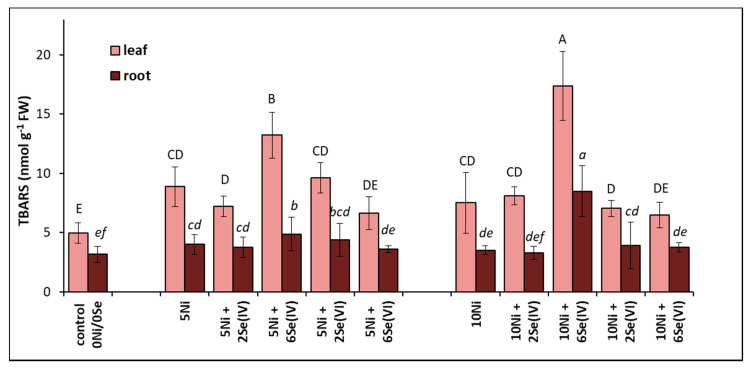
Effect of Se supplementation on the lipid peroxidation (expressed as the TBARS concentration) of Ni-exposed lettuce after 14 days of exposure. Data are means ± SD (*n* = 6). Means for individual organs followed by the same letter are not significantly different (*p* < 0.05, Tukey’s test).

The level of lipid peroxidation increases with increasing concentrations of TBARS in tissues. Plants grown in a medium containing 5 or 10 µM Ni had a 79% or 52% higher level of TBARS in their leaves and a 45% or 28% higher level of TBARS in their roots, respectively, compared to the control (Figure 3). The greatest increase in TBARS accumulation was found in plants treated with Ni and supplemented with 6 µM Se(IV). In these conditions, the foliar TBARS level increased by 51% and approx. 2-fold at 5 or 10 µM Ni, respectively, compared to the Ni-alone treatments. A similar relationship was noted in the roots. In the other treatments, the level of lipid peroxidation in the tissues after Se supplementation did not fluctuate significantly in relation to the Ni-stressed plants (Figure 3).

### 3.3. Effect of Se(IV) and Se(VI) on the Viability of Root Tips in Ni-Exposed Lettuce

**Figure 4:**
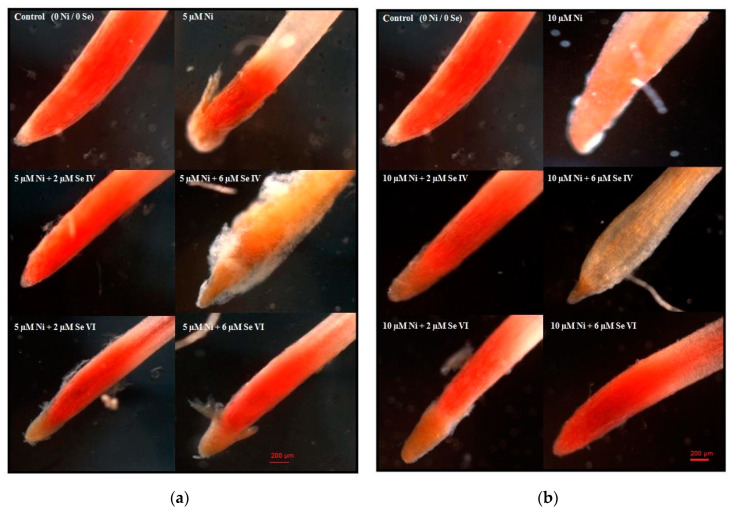
Influence of Se supplementation on the viability of root tips (staining with the 2,3,5-triphenyltetrazolium chloride (TTC) method) of Ni-exposed lettuce after 14 days of exposure. (**a**) Root tips of plants grown at 5 µM Ni and supplemented with Se; (**b**) root tips of plants grown at 10 µM Ni and supplemented with Se.

The intense red–pink root tips are characterized by a high level of cellular respiration and viability; a reduction or absence of the color indicates disturbances in root cell metabolism and a decrease in cell viability. The analysis of the microscopic images (Figure 4a,b) showed that the root tips of the control plants were metabolically vigorous, while the presence of Ni contributed to a reduction of their viability. Moreover, a substantial loss of the activity of root dehydrogenases was observed after the supplementation of Ni-contaminated medium with 6 µM Se(IV) (Figure 4b). In other cases, the viability levels of Ni-stressed roots were generally stimulated by Se addition in comparison to plants grown in the Ni-alone treatments (Figure 4a,b).

### 3.4. Effect of Se(IV) and Se(VI) on the Concentration and Translocation of Ni and Se in Ni-Exposed Lettuce

The application of Se at the concentration of 6 µM to the Ni-contaminated medium increased the accumulation of this metal in the shoots, especially when Se(IV) was added (Table 1). The highest shoot Ni concentration was recorded after the application of 6 µM Se(IV), when its content at 5 and 10 µM Ni increased by 61% and 39%, respectively. Under the influence of Se(VI), a relatively high Se accumulation in the lettuce shoots was also shown. 

Opposite relationships were found in the roots (Table 1). Supplementation of Ni-stressed plants with Se resulted in a decrease in the concentration of this metal in the roots, regardless of the concentration and form of Se. In Ni-exposed plants treated with 6 µM Se(IV), the root Ni amounts decreased by approx. 25% and the TF value for Ni increased. Simultaneously, the root content of Se was very high, which may be the main cause of the increase in Ni phytotoxicity in these conditions. On the other hand, the increase in Ni concentration in the medium did not generally alter Se concentrations in the roots and shoots. 

## 4. Discussion

The experimental Ni concentrations of 5 and 10 µM exceeded the highest permissible level of this micronutrient in groundwater by 12.5 and 25 times, respectively. Simultaneously, the 5 µM concentration of Ni was about 100-fold higher than the background concentration of this element in groundwater and exceeded the global average value by 16 times. However, both these Ni concentrations were lower than the range found for water in industrial regions, which is 0.6–60 µM Ni [24,25]. The second differentiating factor was Se form and concentration. The experimental Se concentrations of both forms used were 2 or 6 µM. The toxicity thresholds for Se(IV) and Se(VI), determined on the basis of growth parameters of the lettuce cv. Justyna, were 15 and 20 μM, respectively [28]. Therefore, Se concentrations below the designated toxicity thresholds with a proven growth-promoting effect for both forms [28] were used in this experiment. 

Reduction of plant growth at toxic Ni concentrations has been documented in numerous experiments as a consequence of disturbances in all physiological processes: water uptake, transpiration, respiration, photosynthesis, uptake and distribution of essential elements, nitrogen metabolism, and many others [4,5,9,19]. This reduction was usually directly proportional to the Ni concentration in the growth substrate [1]. Accordingly, in our research, the application of 5 or 10 µM Ni caused a dose-dependent decrease in plant FW (Figure 1). Previously, we showed that an application of 50 µM Ni into the nutrient solution caused a lower decrease in lettuce FW than that noted in this experiment; however, the previous study involved slightly different experimental conditions and older plants [19]. This indicates a very important factor determining the resistance of plants to Ni, i.e., their age and progress in development. The exposure of plants to Ni also evoked changes in the accumulation of photosynthetic pigments: a reduction at 5 µM Ni and an increase at 10 µM Ni (Figure 2). The reduced level of chlorophyll and carotenoids may be related to Ni-induced cellular disorganization, which may cause agglutination of the chloroplast thylakoid system [32,33]. On the other hand, the increased pigment accumulation noted at 10 µM Ni may have resulted from a reduction of leaf area, lower biomass (Figure 2), and, presumably, lower water content.

In our previous study on Ni-exposed lettuce, Se applied as Se(IV) (5 µM) was beneficial to the plants and stimulated their growth and accumulation of photosynthetic pigments [19]. However, as already mentioned, the plants were more advanced in development (older) than in this experiment. Our current results indicate that the roots of plants treated with 10 µM Ni responded positively to the Se supplementation in terms of growth, but only when Se was applied at the concentration of 2 µM as Se(VI) (Figure 1). Similarly, in other experiments using hydroponics, the growth of roots was stimulated more efficiently by Se than that of shoots in stressed plants [18,34]. Moreover, it was found that under Se supplementation of Ni-exposed lettuce, the levels of photosynthetic pigments were restored to those found in the control plants (Figure 2), which may be related to the Se-induced rebuilding of the chloroplast ultrastructure [35] and/or the enhancement of the chloroplast antioxidant defense system [36]. 

The lipid peroxidation of biomembranes is the most commonly studied symptom of oxidative stress in plants. In this study, the level of lipid peroxidation increased in the leaves and roots of the Ni-exposed lettuce (Figure 3). Likewise, dose-dependent intensification of lipid peroxide accumulation was evident in leaf tissues of Ni-treated wheat [37]. However, the exogenously applied Se did not prevent this harmful process in either the roots or the leaves of lettuce (Figure 3), as shown in other studies [18,38]. It was found that exogenously applied Se(IV) (15 µM), regardless of its beneficial influence on the growth of Ni-treated wheat, did not prevent the negative consequences of Ni stress on root and shoot phospholipid composition [39].

In the Ni-alone treatments and when 6 µM Se(IV) was added into the Ni-containing medium, especially at the higher Ni dose, we observed a significant loss of viability of the root tips. We suppose that the high accumulation of these two trace elements in the roots (Table 1) significantly impaired the metabolism of root cells, which lost their viability. On the other hand, the other Se treatments enhanced the activity of root tip mitochondrial dehydrogenases in comparison to the Ni-alone treatments (Figure 4). A negative effect of Se applied as Se(VI) on the vitality of the root tips of Cd-exposed plants was shown in research on cucumber [18]. However, the roots in these studies were stained using a different method. Dose-dependent enhancement of root cellular respiration (estimated using the TTC method) was noted in cucumber exposed to Se(IV), but the level of lipid peroxidation and the growth-reducing effect of Se increased as well [40]. In turn, the beneficial influence of exogenous malic and acetic acids under Cd exposure was related to increased mitochondrial dehydrogenase activity and root tip viability in sunflower [41]. This indicates that an increase in cellular respiration in the cells of root tips does not have to be associated with a reduction in the toxicity of the tested factors. It can serve as an additional parameter, reflecting changes in the metabolic activity of plants in various experimental conditions.

It was shown that when 6 µM Se, regardless of its form, was applied to Ni-containing substrate, the shoot Ni content increased, whereas the root Ni concentration decreased, for all Se treatments. Similarly, in our previous experiments with older lettuce, the supplementation of plants exposed to 50 µM Ni with Se(IV) (5 µM) enhanced Ni accumulation in the aboveground organs [19]. The apoplast is the main route of Ni uptake and transport in roots [42]. Some studies indicated that exogenous amino acids (histidine, glycine, and glutamine) enhanced the symplastic-to-apoplastic Ni ratio in the roots and stimulated the translocation of this metal to the shoots [43]. It is probable that Se ions or Se amino acids also contribute to the symplastic uptake and further translocation of Ni, but detailed studies in this regard are required to support this statement. Application of 10 µM Se(VI) to substrate contaminated with 50 µM Cd resulted in a decrease in the concentration of this metal in the roots but not the shoots of cucumber [18]. In turn, Feng et al. [44] noted a decrease in Cd accumulation in Se-supplemented paddy rice. However, this effect was largely dependent on the mutual quantitative proportion between Cd and Se ions in the growth medium, and, when the Cd level was high (>89 μM), the addition of Se resulted in an intensification of Cd uptake. 

We found a very high accumulation of this element in the root tissues in the presence of Se(IV), which is consistent with the results of other studies [28,40,45]. In roots, Se(IV) can be rapidly converted to organic Se (e.g., selenomethionine, selenomethionine Se-oxide, Se-methyl-selenocysteine, and several unidentified Se forms), with restricted translocation to shoots and toxic effects on root cell metabolism [46]. This fact may explain the significant intensification of Ni phytotoxicity symptoms in the presence of Se(IV) (Figure 1) when combined with oxidative stress, which is manifested by a significant increase in lipid peroxidation (Figure 3) and an almost complete loss of root viability (Figure 4). 

In the light of the present research, although the stimulating effect of Se at 2 µM Se(VI) on the growth of the roots of 10 µM Ni-treated plants was confirmed, the young lettuce plants reacted very unfavorably to 6 µM Se(IV)—the concentration with proven growth-stimulating efficacy in similar experimental conditions but without Ni addition [28]. Therefore, depending on the conditions in the rhizosphere, Se in a given concentration and chemical form can be both a biostumulant and a toxicant. The obtained results indicate the great importance of the age of plants, rhizosphere conditions, and the chemical form of Se applied as a plant biostimulant or for plant biofortification with this element.

## 5. Conclusions

We conclude that the Se(IV)-mediated enhancement of Ni toxicity is associated with simultaneous high accumulation of Se and Ni in root tissues and Se-induced lipid peroxidation, which can disrupt root cell metabolism and induce a significant decrease in root viability. Therefore, we postulate that when Se is used to alleviate the toxicity of metals that accumulate mainly in the root tissues (Cd, Ni), the form of Se must be taken into account, in particular, because the simultaneous root accumulation of Se applied as Se(IV) and another metal may enhance the toxicity of this metal. Then, although the concentration of Se is well below the toxicity threshold for a given species, as in our studies on lettuce, Se may increase metal toxicity, giving the exact opposite effect to the expected one. On the other hand, in our research, only 2 μM of Se(VI) had a positive effect on the growth of roots. In all the Se treatments, except for 6 μM Se(IV), improved accumulation of photosynthetic pigments and increased viability of the root tips, without significant changes in lipid peroxidation, were found. To sum up, Se can modify Ni phytotoxicity, but this depends not only on the concentration but also on the chemical form of Se. 

## Figures and Tables

**Table 1:** Effect of Se supplementation on Ni and Se concentrations in the shoots and roots of Ni-exposed lettuce after 14 days of exposure.

Concentration of the Element in the Medium (µM)		Concentration of the Element in the Shoots (mg kg^−1^ DW)		Concentration of the Element in the Roots (mg kg^−1^ DW)		Translocation Factor (TF); Shoot/Root Element Content Ratios	
Ni	Se	Ni	Se	Ni	Se	Ni	Se
***0***	***0***	1.12 ± 0.44 h	0.37 ± 0.12 e	1.90 ± 0.12 g	0.41 ± 0.10 h	0.589 ± 0.23 a	0.902 ± 0.11 a
***5***	***0***	12.90 ± 1.65 f	0.42 ± 0.16 e	312.9 ± 35.2 d	0.56 ± 0.12 h	0.041 ± 0.008 f	0.750 ± 0.08 bc
***5***	***2 Se(IV)***	13.30 ± 1.34 f	2.97 ± 0.38 d	268.4 ± 24.8 e	40.40 ± 4.33 c	0.050 ± 0.014 ef	0.074 ± 0.02 f
***5***	***6 Se(IV)***	20.80 ± 2.64 e	31.60 ± 4.88 a	231.2 ± 20.1 f	216.60 ± 28.3 a	0.090 ± 0.012 c	0.146 ± 0.02 e
***5***	***2 Se(VI)***	14.30 ± 1.01 f	6.23 ± 0.75 c	241.3± 21.4 f	8.90 ± 1.36 f	0.059 ± 0.009 e	0.700 ± 0.06 cd
***5***	***6 Se(VI)***	15.80 ± 2.11 g	18.90 ± 2.92 b	263.7± 12.3 e	28.30 ± 3.16 d	0.060 ± 0.005 e	0.668 ± 0.04 d
***10***	***0***	23.70 ± 3.65 d	0.59 ± 0.21 e	426.5± 36.8 a	0.66 ± 0.21 h	0.056 ± 0.007 e	0.894 ± 0.11 a
***10***	***2 Se(IV)***	23.90 ± 3.11 d	2.82 ± 0.38 d	365.5± 22.6 c	62.90 ± 5.25 b	0.065 ± 0.004 cd	0.045 ± 0.02 f
***10***	***6 Se(IV)***	32.90 ± 3.58 a	33.30 ± 5.44 a	320.5± 24.9 d	225.0 ± 17.54 a	0.103 ± 0.013 b	0.148 ± 0.03 e
***10***	***2 Se(VI)***	25.30 ± 2.47 cd	6.34 ± 0.74 c	338.5± 22.7 d	8.50 ± 0.98 f	0.075 ± 0.005 d	0.746 ± 0.09 bc
***10***	***6 Se(VI)***	27.50 ± 3.25 bc	19.50 ± 2.36 b	397.5± 16.6 b	25.70 ± 3.00 d	0.069 ± 0.004 d	0.759 ± 0.04 b

Data are means ± SD (*n* = 3). The means for individual elements and TF in the same column, when followed by the same letter, are not significantly different (*p* < 0.05, Tukey’s test).
